# Vascular Complications of Infective Endocarditis: Diagnosis and Management

**DOI:** 10.7759/cureus.14678

**Published:** 2021-04-25

**Authors:** Kissami Ibtisam, Mehdi El Bekkaoui, Imane Skiker, Yassamine Bentata, Nabila Ismaili, Noha Elouafi

**Affiliations:** 1 Cardiology, Faculty of Medicine and Pharmacy, Centre Hospitalier Universitaire, Mohamed First University, Oujda, MAR; 2 Radiology, Faculty of Medicine and Pharmacy, Centre Hospitalier Universitaire, Mohamed First University, Oujda, MAR; 3 Nephrology, Faculty of Medicine and Pharmacy, Centre Hospitalier Uiversitaire, Mohamed First University, Oujda, MAR; 4 Cardiology, Mohammed I University/Mohammed VI University Hospital, Oujda, MAR

**Keywords:** infective endocarditis, vascular complication, mycotic aneurysms, embolic events

## Abstract

Introduction: The complications of infective endocarditis (IE) are frequent and severe. Our aim was to study the clinical and paraclinical profiles and prognosis of vascular complications, observed in a cardiology unit in Oujda, Morocco. Among 57 patients hospitalized for IE between 2015 and 2020 at the cardiology unit, 19 patients, or 33.3% of patients, had one or more vascular complications. We present here a retrospective analysis.

Aim: Prevention, early diagnosis, and treatment of vascular complications are primordial to improving prognosis, following the guidelines of the European Society of Cardiology.

Patients and methods: We retrospectively studied 57 patients hospitalized for IE. The diagnostic criteria for IE were modified from the Duke University criteria and we present all vascular complications among this cohort.

Results: Nineteen patients presented with one or more vascular complications, 10 men and nine women, with a mean age of 49 years. IE had grafted on a mechanical prosthetic valve in four cases. Overall, we found 25 vascular lesions: six neurological complications, five cases of peripheral vascular involvement, nine splenic infarcts, and five recurrent septic pulmonary embolisms (SPEs). The vascular complications accrued after three to 14 days of antibiotherapy or on extension reports; blood cultures were positive in 17 (89.4%) cases; streptococcus was isolated in nine cases; *Staphylococcus aureus* in seven cases; and acinetobacter in one case.

Conclusion: Vascular complications of IE are severe, the most common in our study being splenic infarct. Prevention and early diagnosis are essential to instituting optimal management.

All the patients were followed up with a mean follow-up of three years. Late mortality involved one patient in connection with a hemorrhagic stroke secondary to an accident with vitamin K antagonists after its release in one month. No cases of recurrence of endocarditis were noted in this group.

Data were collected from archived medical records and analyzed by Statistical Package for the Social Sciences.

## Introduction

Infective endocarditis (IE) is an intravascular infection that is defined as microbial invasion of the endocardium that lines the valves or chambers of the heart [1]. Despite therapeutic progress, complications of IE remain frequent and severe. The global intra-hospital mortality of IE is 20% and reaches 40% after one year of follow-up [2], mostly related to complications. Embolic events in 20%-50% of patients with IE can be underdiagnosed, especially for the splenic or cerebral circulation, and it can be definitively diagnosed by noninvasive imaging. Overall, the occurrence of embolic complications is 20%-50% in patients with IE but falls to 6%-21% after initiation of antibiotic treatment.

The aim of this work is to emphasize the early diagnosis and treatment of vascular complication in order to improve prognosis and determine the organism involved. Despite therapeutic progress, IE remains a real diagnostic and therapeutic problem. When is the optimal time for early surgery and what type of valve can be used? What type of infectious agent is involved and how can it be prevented?

## Materials and methods

We retrospectively studied 57 patients presenting with IE, hospitalized between 2015 and 2020 in the cardiology units of Oujda University Hospital in Morocco. The diagnostic criteria of IE were modified Duke University criteria. We selected for our study population patients with one or several vascular complications, diagnosed on the basis of clinical and/or paraclinical data. The studied parameters were clinical, bacteriological, transthoracic and transesophageal echocardiography, Doppler and data of other paraclinical examinations according to the type of vascular complication (brain CT scan, vascular echo Doppler, and MRI).

The following vascular complications were investigated: neurological vascular complications, arterial complications in the limbs, splenic infarction, renal infarction, abscess, and pulmonary embolism.

Data were collected from archived medical records and analyzed by IBM®, Armonk, New York.

## Results

The study included 57 cases of IE, 19 of which had at least one vascular complication. The frequency of these complications was thus 33.3%. The mean age was 49 years (ranging from 16 to 82 years) and the sex ratio of male/female was 1:1. The etiology was dominated by the dental in nine cases followed by the cutaneous pathways in four cases. No etiology could be demonstrated in six cases. Vegetations were found in 15 cases; observed on the mitral valve in seven cases, the aortic in four cases, and the tricuspid in five cases. Multiple locations were found in four cases. Blood cultures were positive in 15 cases. The most-identified organism was streptococcus in nine cases and one case of death due to tricuspid endocarditis complicated with septic shock.

Treatment consisted of appropriate antibiotic therapy, secondarily rehabilitated according to blood cultures data and urgent surgery for definitive management of vascular complications. The choice of which valve is difficult, taking into consideration the hemorrhagic risk inherent in mechanical valves, and the bioprosthetic valves, which should have a lifespan greater than that of the patient.

Neurological complications

**Figure 1:**
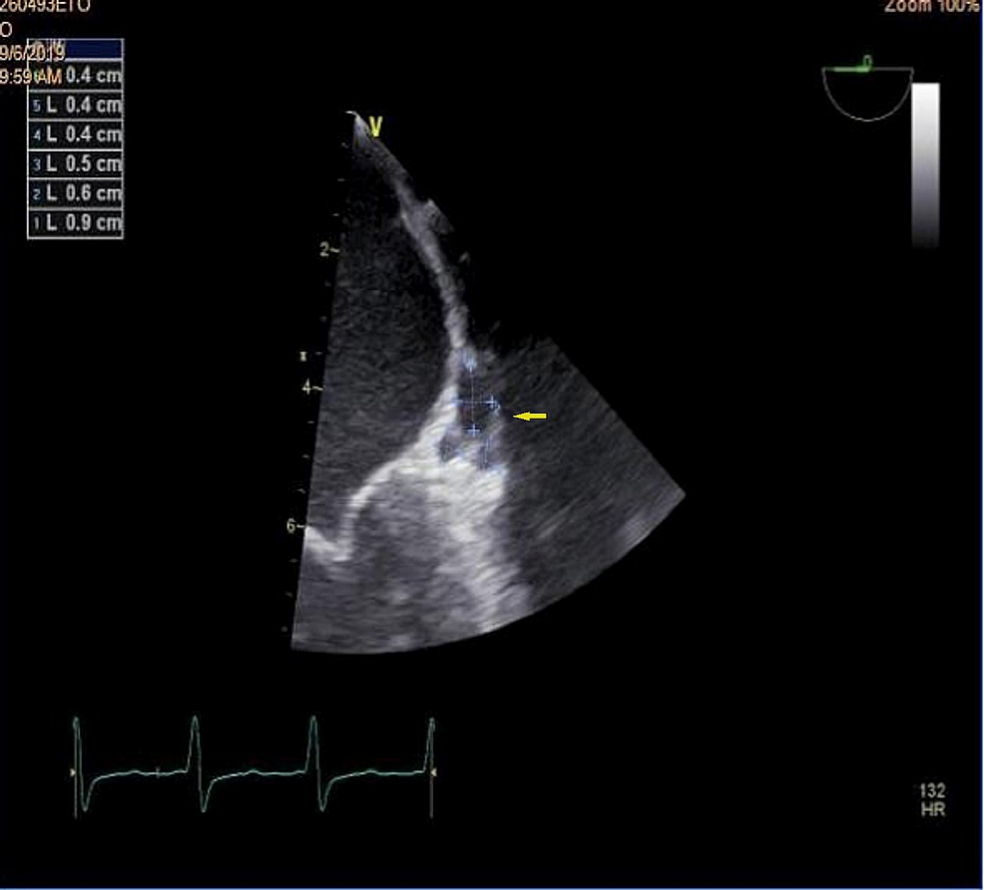
Presence of three abscesses in the aortic ring on echocardiography.

**Figure 2:**
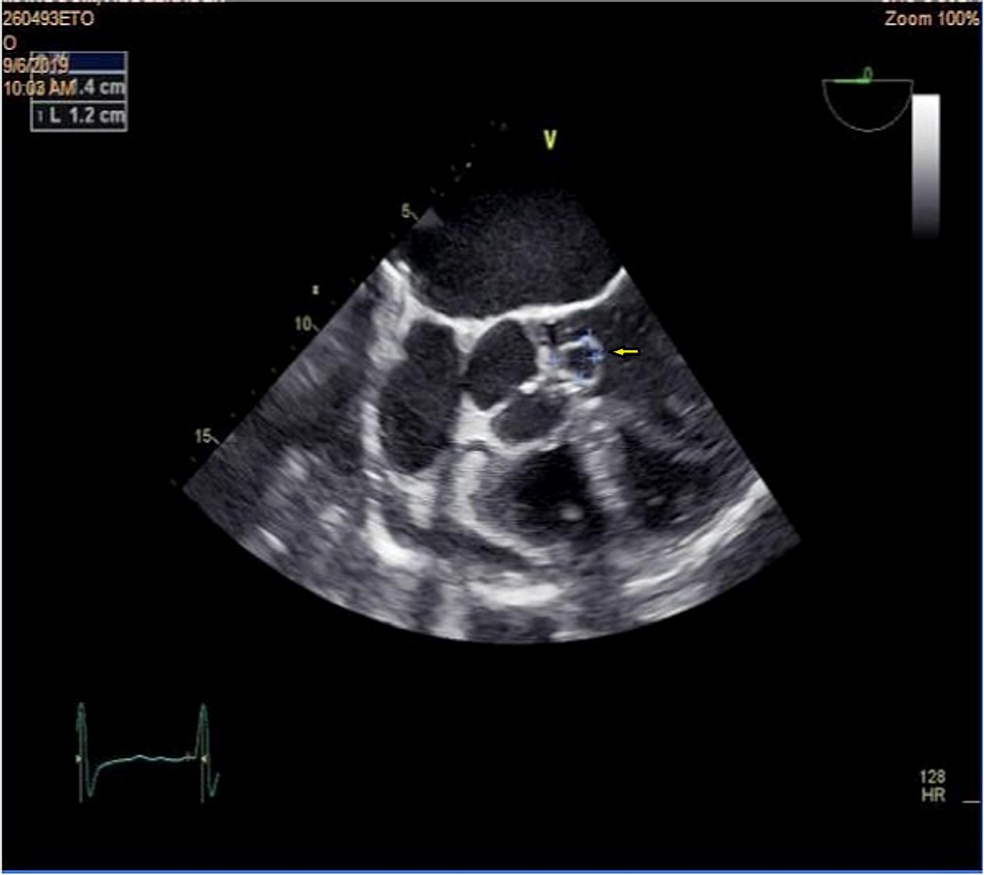
Aortic growth at the expense of the left coronary cusp.

**Figure 3:**
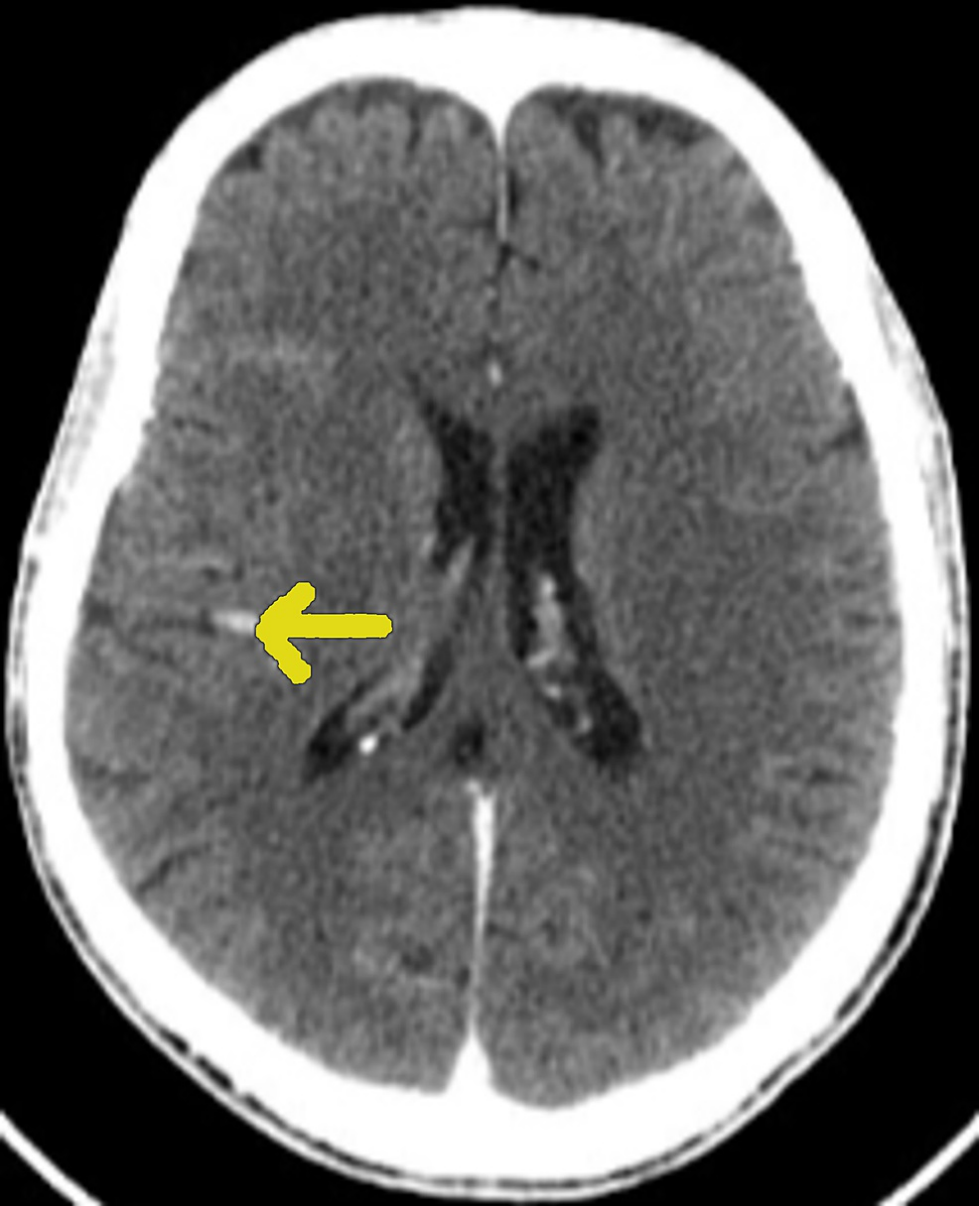
Cerebral CT image of an axial section showing a cerebral mycotic aneurysm.

Six neurological complications were observed. The clinical pictures produced by these neurological complications are polymorphic: one case of hemiplegia, two cases of epileptic seizure, and three cases of confusion and agitation. Four out of six patients were on anticoagulants and the site of the IE was mitral-aortic (Figures 1-2) for all cases. We have thus identified: three mycotic aneurysms (Figure 3), two ischemic cerebrovascular accidents, and one intracerebral hematoma. Diagnosis was provided by brain scan and MRI. Neurosurgical treatment was not performed in our patients because of the small size of the lesions. The infectious agent identified was staphylococcus in three cases and streptococcus in one case. The choice of the prosthesis depends on the age and on the possibility (or not) of anticoagulation. For our series, only one patient of 50 years, complicated by a lacunar stroke, underwent a mechanical valve replacement with lifelong anticoagulation. For another, 68 years old and complicated by a mycotic aneurysm and intra-cerebral hematoma replacement, replacement of the aortic valve by bio-prosthesis and a mitral annuloplasty were carried out; vitamin K antagonist anticoagulant was maintained for three months with good progress. The outcome of this group of patients was reassuring and all patients retained motor sequelae during hospitalization and within six months to one year after.

Peripheral complications

Septic Pulmonary Embolism (Five Cases)

**Figure 4:**
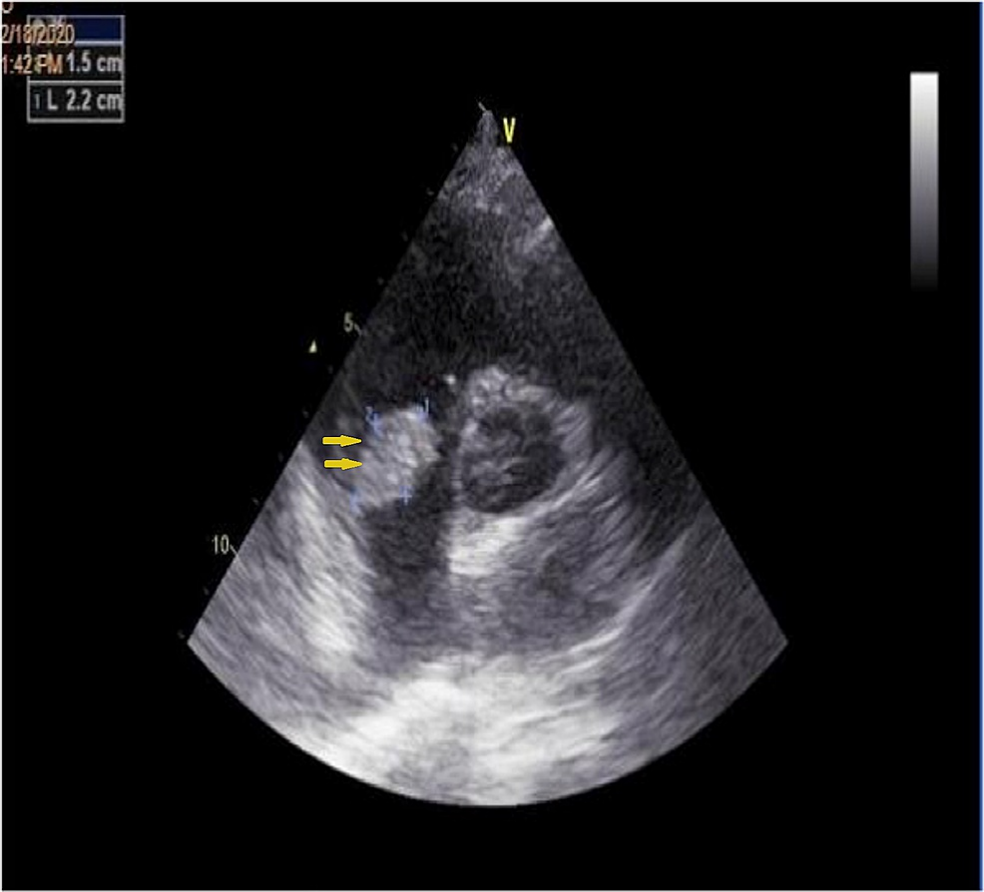
The short parasternal axis reveals bulky vegetation on the tricuspid valve.

**Figure 5:**
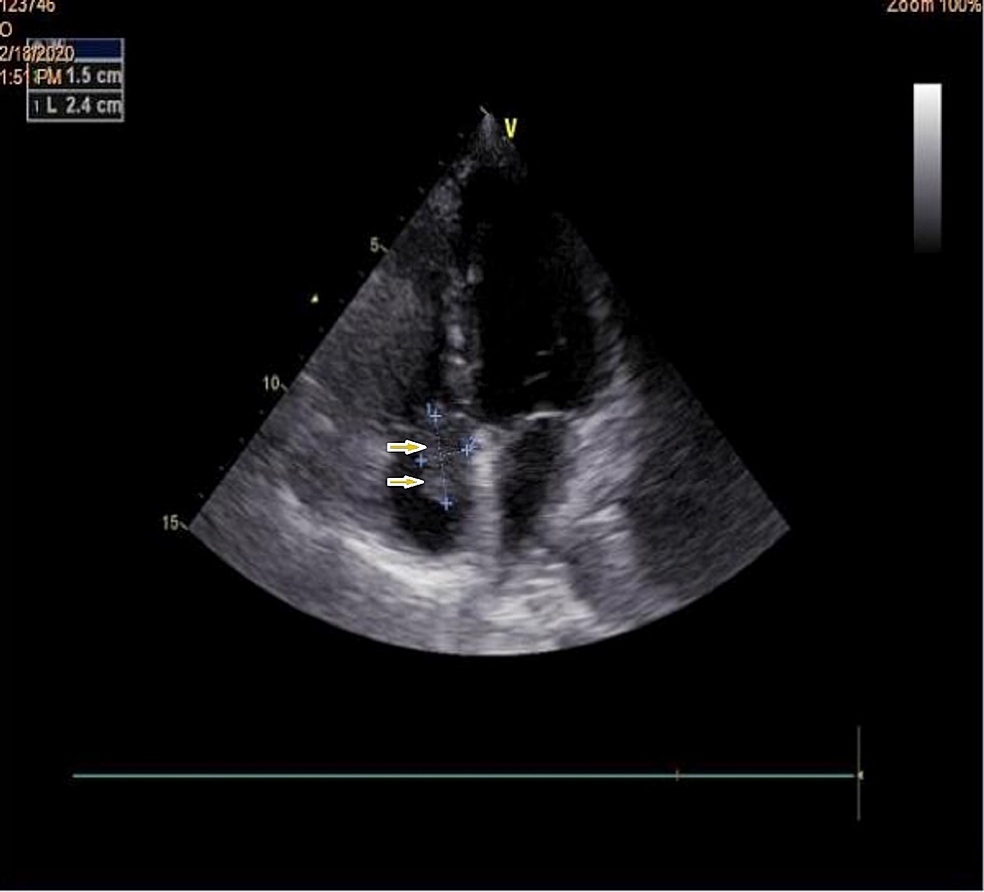
Apical section of four cavities showing the tricuspid mass on echocardiography.

**Figure 6:**
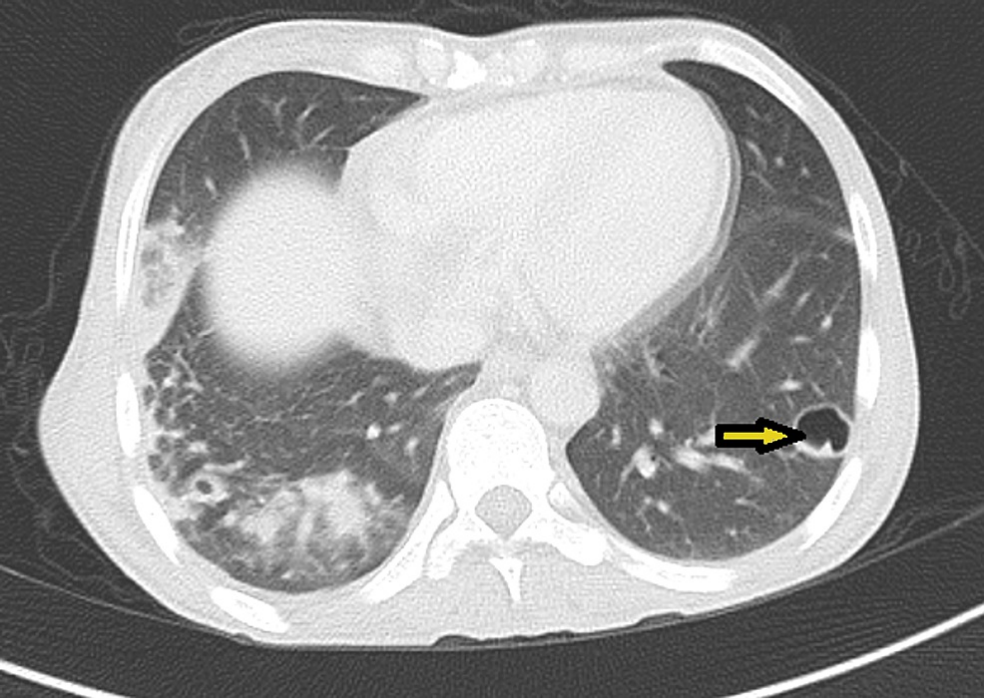
Thoracic CT scan of an axial slice showing pulmonary septic embolism.

This was noted during the course of tricuspid infection endocarditis in five cases (Figures 4-5). The diagnosis was made in the presence of left basi-thoracic pain and fever in two presentations, with individualization of the SPE on CT scan (Figure 6). Streptococcus was the dominant organism in three cases followed by acinetobacter and staphylococcus in each case. The outcome under appropriate medical treatment was favorable except one patient who died from septic shock.

Splenic Infarction and Abscess (Nine Cases)

**Figure 7:**
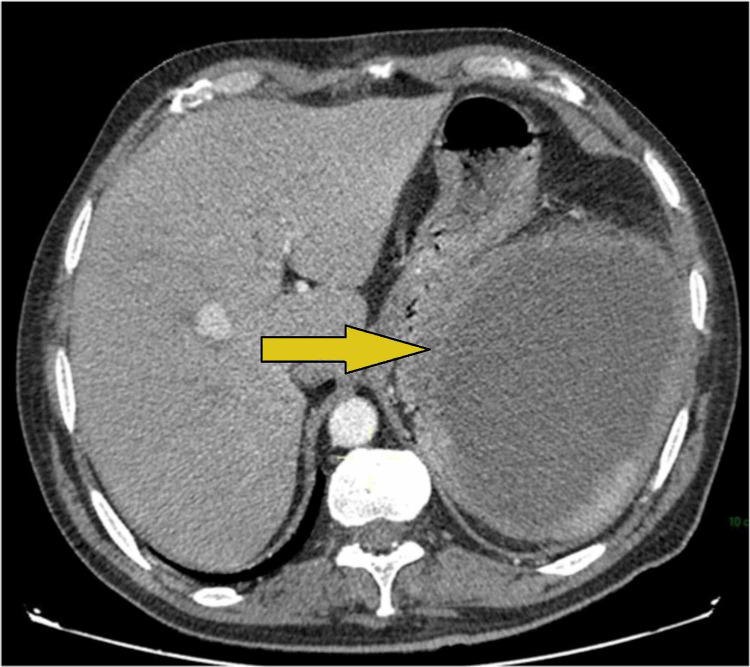
Axial section of abdominal CT scan showing a splenic abscess.

The diagnosis was made by the finding of a painless splenomegaly during treatment in three patients including seven IE of mitral origin. This diagnosis was confirmed by ultrasound in three patients and the abdominal CT scan (Figure 7) during the extension assessment in the other patients. In this case report, a splenic abscess complicating endocarditis was cured by antibiotic treatment in six cases, splenectomy for two patients, and drainage of a splenic abscess for another one because of the resistance to antibiotic therapy and the large size of the abscesses. The streptococcus and the staphylococcus were the leading germs in cause but in four cases no germ has been identified and only one replacement of the mitral valve was made.

Arterial Involvement of the Limbs (Five Cases)

Aortic endocarditis was the main cause in two cases followed by mitral-aortic in three cases, mainly due to staphylococcus in four cases, including an aneurysm of the brachial artery revealed by ischemia of the upper limb that benefited from flattening and grafting of the artery. The aneurysms of the popliteal artery, the ileal artery, and the right and the left femoral artery have been respected due to the insignificant sizes.

## Discussion

Neurological complication

Cerebral complications are the most frequent of the extra cardiac complications of IE. Despite recent advancement in tools for diagnosis and medical therapy, as well as surgical procedures, there remains a high rate of morbidity and mortality [3]. Their prevalence is between 10% and 65% according to imaging studies [4-5]. With neurological complications mortality can reach 50% [6-7]. Our study presents a rate of 24% for neurological complications, among them three cases of stroke and three mycotic aneurysms. Stroke is the most common neurological complication of IE and complicates 20%-40% of all cases. Embolism of a vegetation fragment or a rupture of an intracranial mycotic aneurysm are the two ways in which stroke complicates IE [5] and can be asymptomatic. The clinical consequences are related to the size of the emboli [8]. Neurological imaging can be helpful to detect patients with asymptomatic neurological complications and to identify patients in whom neurological complications may influence further therapeutic decisions. There is insufficient information as to whether all patients with IE, irrespective of neurological symptoms, should undergo brain imaging with MRI or at least with cranial CT, to screen for asymptomatic embolism and to exclude intracerebral hemorrhage [9]. Although there are no studies directly comparing CT and MRI for the evaluation of neurological complications of IE, MRI discloses lesions not visible on CT, including embolic infarction, abscess, and subtle petechial hemorrhage. We identified a few risk factors: first, the vegetation size. Di Salvo et al. investigated 178 patients with IE of whom 37% had one or more embolic events. In patients with vegetation length >10 mm, the risk of embolism was 60% in contrast to those in whom the size of the vegetation was <10 mm when the risk of embolism was 23% (p = 0.001) and there was an increasing rate of mortality [10]. Second, the mobility of vegetations: Depréle and al. found that in patients with mobile vegetations, the risk of an embolic event was 48% in contrast to fixed vegetations where the risk was 9% [11]. On the other hand, the pathogen also carried a different risk; in a recent meta-analysis of 11215 cases of IE, Staphylococcus aureus infection was associated with the highest rate of embolic events taking place [12], which aligns with the results of our study.

Hemorrhagic stroke may be explained by three main mechanisms: the hemorrhagic transformation, the rupture of an intracranial mycotic aneurysm, and the rupture of an intracranial vessel due to necrotizing arteritis [13].

The diagnosis of IE causing embolic accident contraindicates thrombolysis due to high hemorrhagic risk [14]. Mechanical thrombectomy has been recently proposed successfully [15]. Brain MRI is the recommended examination for the detection of symptomatic and asymptomatic neurological complications. The occurrence of symptomatic neurological complications obviously influences the therapeutic management, and extensive lesions in severely symptomatic patients are a contraindication to heart surgery and we question whether we can put anticoagulants. When to operate? Which valve to choose? For one of our patients, we elected surgery after two weeks of antibiotherapy with a bioprosthesis in view of advanced age, comorbidity, and the presence of mycotic aneurysm and intracerebral hematoma. The other patient, who was young with a low hemorrhagic risk, received a mechanical valve replacement, which adheres to the recommendations of the European Association of Cardio-thoracic Surgery, published in 2017, but the timing of the surgery is still unclear.

Peripheral complications

Septic Pulmonary Embolism

Septic pulmonary embolism (SPE) is a type of nonthrombotic pulmonary embolism. The right cardiac vegetation repeatedly falls off and enters the pulmonary artery and its branches, causing a pulmonary infarction and metastatic abscesses [16]. Pulmonary embolisms occur in 47.8% of cases of right-sided IE, and lung abscesses occur in 15.5% [17]. Predisposing factors for SPE in the setting of IE include IV drug use, valvular heart disease, congenital heart disease, and the presence of implanted cardiac devices or placement of central lines or catheter [18]. The streptococcus and the staphylococcus are the most common isolated pathogens, thus joining the results of our study. X-ray images and trans-thoracic echocardiography data confirm the diagnosis. Pulmonary valve replacement surgery, in the absence of right heart failure, should not be performed, according to the Guidelines for the Management of Infective Endocarditis. For our study we observed better therapeutic responses to medical treatments with antibiotics.

Splenic Infarction and Abscess

These are often asymptomatic. Anatomically, the lesions are usually multiple and result from obliteration of one of the branches of the splenic artery or their ramification. Indeed, splenic abscess or infarction may be a disease entity at different stages in patients of IE due to septic emboli of the spleen. The treatments of choice are antibiotics, splenectomy, and valve replacement; these should be individualized according to the extension and location of the abscess and/or infraction. The indications for the surgical treatment of IE reported in the literature are similar to those in our study.

Arterial Involvement of the Limbs

This has a frequency of 20%-30%. The involvement of the lower limbs is more frequent than the upper limbs. They rarely involved vital prognosis; on the other hand, the functional one is compromised in the event of a diagnostic or therapeutic delay. First the diagnosis is clinical and then confirmed by CT angiography of limbs. However, surgical intervention is the treatment of choice following long-term medical treatment. This is consistent with the conduct of other studies in the literature.

## Conclusions

Vascular complications of IE are frequent and severe. Their screening is essentially based on a complete and repeated clinical examination and their prevention requires early and correct treatment. Despite the therapeutic advances, this remains a real challenge. Other studies will be necessary to make specific recommendations.
